# Nature Studies.—VI

**Published:** 1910-06-25

**Authors:** 


### The Practitioner's Relaxations.

### NATURE STUDIES.—VI.

By A MAJOR R.A.M.C.

A Valley in the Downs—A Seventh Year Flood-time—Local Tradition—The Rise of a Stream—Unsuspected Levels—Under-gravel—Winterbournes.

During the last few months I have been watching an interesting happening.

My house is 300 feet above the sea, in a narrow valley that runs north and south through the downs. The head of this valley is about ten miles to the north, it ends as far to the south. On either side the down rises steeply till, two and a half miles behind my abode, the greatest height is over 600 feet. It is nearly as great at other points on either side the valley. In such a valley there should be a river, and so there is; but no ordinary river. There is generally water in the river six miles to the north of my house; six miles to the south there is always water; but the intervening twelve miles is often more or less dry. That part which begins three or four miles north of my house and ends two miles below it is usually dry.

The only traces of a river when I came here early last autumn was a dry ditch, some 8 feet wide, choked with a thick growth of docks, grass, and nettles. Now there is a little river flowing rapidly. According to local tradition this river comes down every seventh year. But seven is a number loosely used; there is not regular periodicity.

Throughout the winter the village augurs have prophesied that the river would come down this year, but they prophesied from signs—the rising of certain wells, the flowing of certain springs—not from lapse of time. I am told this is the sixth year since the river last came down.

Local tradition also holds that when the river comes down fever and diphtheria visit the villages in the valley. No doubt the explanation is that when the water rises cess-pits and wells exchange contents. But the people here should be safe, for the squire is a practical sanitarian. High up the down, at great cost, he has dug wells in the chalk that provide a pure water-supply. This is pumped up by wind pumps and piped to his village.

I have been watching the coming of the river with interest. It has not come down to us in a sudden flood, but gradually, yard by yard almost. Eight miles north the river has been running all the winter. About New Year's Day I found it running through a village six miles away and spreading out over some small water meadows south of that village. A fortnight later it was running through the village four miles north of my house. It was running strongly, bank high; in places it had overflowed its banks and was spreading over the fields. Once, quite unexpectedly I found the water in February flowing through the village only a mile away and taking up the whole of the road in parts. Perhaps I should mention that the villages in the valley are built right on the river. In many of them parts of the river bed, when dry, are used as roads. A few days later a considerable stream was running half a mile above my house. A week later water was flowing fast just across the road from me, and 400 yards lower down pools of water were forming in the river bed. A few days later the river was running full stream throughout its length.

A villager told me one day that if the river came there would be a lake in front of my house. This I scarcely accepted, for my house faces a great clover field that slopes to the road and appeared to me to be higher than the river in all its extent. But the eye is easily deceived about levels. I did not observe that there is a shallow depression in the north-east corner of the field a good deal lower than the surrounding ground. One day soon after the river had got past my house, I saw a small pool of water about a yard in diameter in this depression. Day after day the pool grew steadily larger, till, on March 18, it attained its maximum, and I had a little lake some 20 yards long and 15 yards across just over the road from my house.

And while the lake was forming water rose in certain gravel-pits that up to this time had been dry; so much came into them that the men working the gravel had to stop work. This gave me a solution of the puzzle.

In the bottom of that part of the valley in which the river is usually dry there is a narrow deposit of gravel that is slightly worked for local needs. No doubt the river is always flowing down the valley, but in normal years it is flowing out of sight down in the gravel. When the chalk hills are saturated by excessive rainfall, such as 1909 has given, then the hidden stream floods and flows above ground as a little river, and its waters rise into the gravel-pits and come up through a pocket of gravel to form the little lake in the depression in the clover field. As the excess of water is drained from the chalk my lake will sink away, and the river will dwindle till it disappears, to run again a hidden course, and not again to show itself till another soaking year. Already there are signs of this happening. Since March 18 the lake has been receding from its high-water mark and the river has dropped four inches.

There are other streams of a similar nature in all parts of the downland. A glance at a map will show many "Winterbournes" marking the course of streams that only flow in the wetter half of the year.

On Salisbury Plain there is "Nine Mile River," near Bulford, marked on the maps as "often dry"; it is always dry in summer in its upper half.

Again, amongst the downs west of the Avon there is Water Dean Bottom, a deep hollow. Along its length there are wells dug for the watering of the sheep. A few weeks ago a shallow river, only a few inches deep but several feet wide, was flowing along the lines of the wells over the down turf.

